# The neoepitope landscape in pediatric cancers

**DOI:** 10.1186/s13073-017-0468-3

**Published:** 2017-08-31

**Authors:** Ti-Cheng Chang, Robert A. Carter, Yongjin Li, Yuxin Li, Hong Wang, Michael N. Edmonson, Xiang Chen, Paula Arnold, Terrence L. Geiger, Gang Wu, Junmin Peng, Michael Dyer, James R. Downing, Douglas R. Green, Paul G. Thomas, Jinghui Zhang

**Affiliations:** 1 Department of Computational Biology, St Jude Children’s Research Hospital, Memphis, Tennessee 38105 USA; 2 Department of Oncology, St Jude Children’s Research Hospital, Memphis, Tennessee 38105 USA; 3 Department of Structural Biology, St Jude Children’s Research Hospital, Memphis, Tennessee 38105 USA; 4 St Jude Proteomics Facility, St Jude Children’s Research Hospital, Memphis, Tennessee 38105 USA; 5 Department of Pathology, St Jude Children’s Research Hospital, Memphis, Tennessee 38105 USA; 6 Department of Developmental Neurobiology, St Jude Children’s Research Hospital, Memphis, Tennessee 38105 USA; 7 Department of Immunology, St Jude Children’s Research Hospital, Memphis, Tennessee 38105 USA

**Keywords:** Epitopes, Pediatric cancer, Immunotherapy, Gene fusions

## Abstract

**Background:**

Neoepitopes derived from tumor-specific somatic mutations are promising targets for immunotherapy in childhood cancers. However, the potential for such therapies in targeting these epitopes remains uncertain due to a lack of knowledge of the neoepitope landscape in childhood cancer. Studies to date have focused primarily on missense mutations without exploring gene fusions, which are a major class of oncogenic drivers in pediatric cancer.

**Methods:**

We developed an analytical workflow for identification of putative neoepitopes based on somatic missense mutations and gene fusions using whole-genome sequencing data. Transcriptome sequencing data were incorporated to interrogate the expression status of the neoepitopes.

**Results:**

We present the neoepitope landscape of somatic alterations including missense mutations and oncogenic gene fusions identified in 540 childhood cancer genomes and transcriptomes representing 23 cancer subtypes. We found that 88% of leukemias, 78% of central nervous system tumors, and 90% of solid tumors had at least one predicted neoepitope. Mutation hotspots in KRAS and histone H3 genes encode potential epitopes in multiple patients. Additionally, the ETV6-RUNX1 fusion was found to encode putative neoepitopes in a high proportion (69.6%) of the pediatric leukemia harboring this fusion.

**Conclusions:**

Our study presents a comprehensive repertoire of potential neoepitopes in childhood cancers, and will facilitate the development of immunotherapeutic approaches designed to exploit them. The source code of the workflow is available at GitHub (https://github.com/zhanglabstjude/neoepitope).

**Electronic supplementary material:**

The online version of this article (doi:10.1186/s13073-017-0468-3) contains supplementary material, which is available to authorized users.

## Background

Cancers are caused by somatically acquired alterations, including single nucleotide variations (SNVs), small insertion/deletions (indels), translocations, and other types of rearrangements. The genes affected by these mutations may produce altered proteins, some of which may lead to the emergence of tumor-specific immunogenic epitopes. While the neoepitopes generated from missense mutations have been investigated extensively [1–4], the immunogenicity of epitopes generated from other types of somatic alterations has remained largely unexplored until recently; now new methods, such as INTEGRATE-Neo [5], are being developed to support gene fusion-derived neoepitope discovery. Neoepitopes presented on the cell surface by major histocompatibility complex (MHC) molecules can be recognized by T cells and elicit immune responses. These may serve as important determinants in the natural immune response to cancer, and are potentially important targets for immunotherapy.

A key factor for antigen presentation and T-cell activation is the binding stability of the peptide–MHC complex at the cell surface. The affinity of an epitope for its cognate MHC molecule is typically measured by its IC_50_ value, where a lower value corresponds to a higher affinity. Previous analyses [1, 6–8] have suggested that an IC_50_ value ≤ 500 nM generally indicates moderate to high affinity of a peptide for MHC, while an IC_50_ value > 500 nM indicates low affinity. Based on machine learning approaches [9], computational algorithms have been developed for prediction of MHC class I peptide binding affinity, enabling a more comprehensive and systematic analysis of immunogenic mutations [10–14]. The accuracy of these approaches varied by the training data used to characterize the binding specificity of the MHC molecules. Consensus approaches combining two or more methods can increase the prediction accuracy when compared with empirical data [13, 15].

Preclinical studies in mice and humans have demonstrated that mutated tumor neoantigens can be recognized by cytotoxic T cells and anti-tumor responses can be induced by immunization with synthetic tumor-specific peptides [16–22]. Mounting clinical evidence has also shown that the neoepitope-specific T cells are important and effective in tumor rejection mediated by adoptive transfer of autologous tumor-infiltrating lymphocytes (TILs) or by immune checkpoint inhibitors [23–28].

As part of the St. Jude/Washington Pediatric Cancer Genome Project (PCGP) we have characterized > 1000 pediatric cancer genomes by whole-genome or whole-exome sequencing [29]. The results have revealed a high variability of somatic mutation rate in different tumor types, ranging from 7.30 × 10^−8^ per base in infant acute lymphoblastic leukemia (ALL) to 1.32 × 10^−5^ per base in pediatric melanoma [30]. Furthermore, we found that somatic alterations resulting in gene fusion represents a major class of oncogenic drivers in pediatric cancer. The genomic heterogeneity of pediatric cancer would require a comprehensive analysis of the neoepitope landscape of pediatric cancer to gain knowledge and insight into the feasibility of employing immunotherapy targeting cancer-specific neoepitopes in this patient population.

**Fig. 1:**
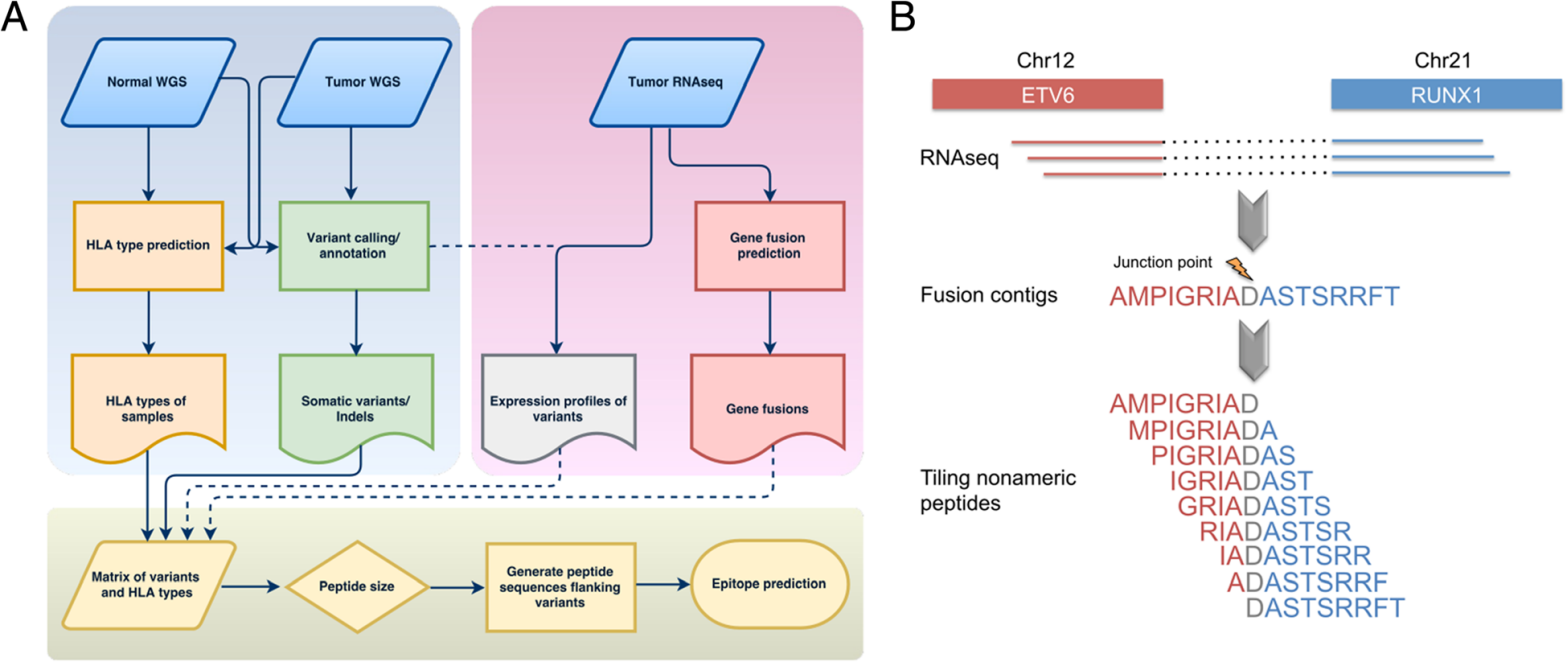
Workflow for HLA typing and neoepitope prediction using WGS and RNA-seq. **a** Overview of analytical process. Somatic missense SNVs for each tumor are identified and annotated based on variants in the aligned WGS data. Gene fusions and expression status of the identified somatic SNVs are analyzed using RNAseq data. All the information is incorporated into a data matrix containing the HLA type, mutation class, amino acid change, protein gi number, mRNA accession number, mutant read count in the tumor, total read count in the tumor, mutant read count in the normal sample, total read count in the normal sample, and reference allele and mutant allele for variants in each sample. The peptide sequences flanking the variations are subsequently extracted and used as input for epitope prediction. **b** Identification of fusion junction peptides at the fusion breakpoints for epitope prediction. An example of ETV6-RUNX1 fusion in SJETV002_D is shown to illustrate this process. Expressed junction reads are assembled from RNAseq. Peptide sequences along the junction position are generated for in-frame coding regions. The tiling nonameric peptides overlapping the fusion breakpoints are subsequently used for epitope prediction

In this study, we characterized the neoepitope landscape of 23 subtypes of pediatric cancer analyzed by whole-genome sequencing (WGS) as part of the PCGP. We developed an analytical process (Fig. 1) for identifying putative neoepitopes based on somatic alteration in a tumor genome and patient’s MHC class I alleles (HLA-A, -B, and -C) using WGS data. These MHC class I alleles encode proteins presenting antigens to CD8^+^ cytotoxic T cells to elicit immune responses, which is essential for eliminating transformed and tumorigenic cells. Importantly, mutant peptides identified through our analysis included those arising from gene fusions as well as missense mutations. Transcriptome sequencing (RNA-seq) data were incorporated into our assessment to identify expressed peptides that can serve as potential candidates for immunotherapy.

## Methods

### Patients, samples, and data

Tumor and matched normal samples were both sequenced in all cases. Matched normal samples were obtained either from peripheral blood, bone marrow, or adjacent normal tissue. Cancer samples were labeled using the following abbreviations: SJACT, adrenocortical tumor; SJAMLM7, acute myeloid leukemia M7; SJCBF, core binding factor acute myeloid leukemia; SJEPD, ependymoma; SJHGG, high grade glioma; SJHYPO, hypodiploid acute lymphoblastic leukemia (ALL); SJINF, infant ALL; SJLGG, low-grade glioma; SJMB, medulloblastoma; SJMEL, melanoma; SJOS, osteosarcoma; SJRB, retinoblastoma; SJRHB, rhabdomyosarcoma; and SJTALL T-lineage ALL. A paired-end WGS strategy was employed for all samples. The sequencing, alignment against human reference genome using BWA [31, 32], and the identification and validation of somatic alterations including missense mutations and gene fusions were described previously [33, 34]. Paired-end reads were aligned against the HG18 or HG19 genome builds depending on when the data were generated.

### HLA typing and WGS validations

The default settings of Optitype were used for HLA analysis. HLA haplotypes derived from WGS were compared with those derived by clinical HLA typing using classic methods (e.g., sequence-specific oligonucleotides, sequence-specific primers, and Sanger sequencing) for 51 patients. All HLA assignments were high resolution per American Society of Histocompatibility and Immunogenetics and College of American Pathologists criteria at the time they were tested. Samples included in this study were tested between 2003 and 2017. For the earliest HLA typing in this set of samples, HLA assignments were made from high resolution sequence-specific primers (SSP; Life Technologies). Sequence-based typing used AlleleSEQR HLA typing kits (Abbott-Molecular) followed by capillary sequencing on an ABI 3130xL or 3500xL genetic analyzer (Life Technology) and analysis using Assign (Connexio Genomics) software. Sequences were compared to sequence-specific oligonucleotide (SSO) typing using LabType bead array test kits (One Lambda) analyzed using the LabScan200 bead array multiplex analyzer (Luminex) and HLA Fusion software (One Lambda). Ambiguities were resolved by sequence-specific primer PCR using SSP primer kits (Life Technologies).

Validation of WGS-based HLA typing was accomplished by comparisons with the clinical HLA Typing validation set. Accuracy was calculated based on the number of correct alleles at the HLA-A, HLA-B, and HLA-C loci. Homozygous loci were counted as two correct alleles if correctly called as homozygous, or one correct allele if it was called as heterozygous with one matching allele.

### Haplotype correlation between HLA and population ethnicity

The HLA alleles called by Optitype were used to infer the HLA haplotypes in each patient using haplo.stats [35]. The haplotype with the highest posterior probability was assigned to each patient. The HLA haplotype frequency in European, African, and east Asian populations was collected from Maiers et al. [36] to compare with the population structure inferred based on the SNP-based genotyping of the 540 patients and SNP data from the public 1000 Genomes (1KG) Project [37]. For the 1KG cohort, we included the SNP data (phase 3) of 299 unrelated individuals with European ancestry (91), African ancestry (105), and East Asian ancestry (103). SNPs on the autosomes were included, and those with the following criteria were excluded: (1) missing genotype rate > 5%, (2) minor allele frequency < 0.01, and/or (3) Hardy–Weinberg *p* value < 0.005. A single SNP was selected per 700 kb on each chromosome. The final dataset contained 3418 SNPs for the 839 individuals. The Admixture model of STRUCTURE v2.3 [38] was run 20 times (20,000 Monte Carlo Markov chain iterations after a burn-in of 10,000 iterations) using default settings and was supervised by the reference population information. The analyses with K =3 maximized the model probability and generated the highest consistency of clustering by assigning membership coefficients to all samples. CLUMPP [39] was used to collate replicate runs and calculate means of fractions of ancestry for each individual. The correlation between the HLA haplotype frequency and SNP-based population structure was evaluated by canonical correlation analysis.

### Neoepitope prediction, RNA expression analysis, mutation signature analyses, and proteomics

Putative neoepitopes were identified by extracting a peptide covering nine tiling nonamers overlapping each missense mutation. Fusion proteins were identified in RNAseq using CICERO [34] (Li et al., unpublished data). Neoepitopes were predicted by obtaining the peptide sequence covering tiling nonamers overlapping each junction (Fig. 1b). NetMHCcons v1.1 [15] was used to predict the affinity of each nonamer for each HLA receptor predicted in each sample. Nonamers were selected if the predicted IC_50_ ≤ 500 nM.

A subset of the patients (*n* = 270) had corresponding RNAseq data [33], which was used to identify the subset of predicted neoepitopes that are expressed. Expression was measured by counting the number of RNA-seq reads supporting the mutant variant, further requiring that at least one of the reads spans the full 27 bases encoding the nonameric peptide.

Mutation signature analyses were performed based on the mutation profiles for eight samples with mutations in the DNA mismatch repair genes or with a high mutation burden. WTSI Mutational Signature Framework was used for the mutation signature analyses [40].

Xenograft mouse models for three rhabdomyosarcoma (SJRHB011_E, SJRHB012_D, and SJRHB026_S) were used to assess whether the expression of neoantigenic transcripts would be a reliable metric for the presence of the mutant peptide. Briefly, proteomics data were generated by two-dimensional LC/LC-MS/MS (Stewart et al., unpublished data) and analyzed by the proteogenomics software JUMPg. [41] Specifically, a customized protein database was generated by translating flanking regions (±30 amino acids) of non-synonymous mutations, which was then concatenated with UniProt human and mouse proteins. MS/MS data were searched against the combined customized amino acid database using the hybrid search engine JUMP [42] and filtered to achieve 1% protein FDR. Spectra exclusively matching to mutation peptides were then manually examined and annotated.

## Results

### Patient cohort

Our cohort consisted of 540 pediatric cancer patients representing 23 subtypes including leukemia (*n* = 284), central nervous system tumors (CNS; *n* = 123), and non-CNS solid tumors (*n* = 133) (Table 1). Relapsed tumors from 18 patients including nine leukemias, five CNS tumors, and four solid tumors were also analyzed. Both the primary tumors and their matching germline samples were analyzed by WGS at 30× coverage. In addition, RNA-seq for 282 tumor samples (270 primary and 12 relapse tumors) were used to interrogate potential neoepitope expression status (Additional file 1). Four high grade glioma (SJHGG003_D, SJHGG030_D, SJHGG034_D, and SJHGG111_D) previously identified as hypermutators [43] were analyzed as a separate group for comparison. We also analyzed cutaneous melanoma (SKCM; *n* = 133), lung adenocarcinoma (LUAD; *n* = 129), and lung squamous cell carcinoma (LUSC; *n* = 33) data acquired from The Cancer Genome Atlas (TCGA) (Table 1; http://cancergenome.nih.gov/) using the same analytical process (Table 1). These three TCGA tumor types known to be susceptible to checkpoint blockade therapies due to high mutation burden [44] were used for comparisons with the results obtained from the pediatric cohort.

**Table 1:** Summary of neoepitope landscape in the PCGP cohort

Project	Class	Disease	Patient number	Sample number^a^	Average number of mutations^b^	Average number of neoepitope (≤500 nM)^b^	Average number of expressed neoepitopes^b^
PCGP	LEUKEMIA	ETV	49	56 (7)	11.22 (20.73)	4.29 (9.25)	1.68 (4.09)
		HYPER	53	53	9.49	4.11	-
		BALL	31	31	11.58	5.03	2.00
		HYPO	22	22	9.64	3.23	1.50
		TALL	10	10	8.10	3.60	-
		ERG	25	25	8.40	3.16	1.28
		CBF	16	16	6.38	2.13	0.89
		INF	19	21(2)	2.47 (3.57)	1.11 (1.52)	0.44 (0.44)
		PHALL	35	35	4.49	1.83	0.52
		E2A	21	21	5.48	2.10	-
		AMLM7	3	3	2.67	0.67	0.33
		Subtotal	284	293			
	CNS	HGG	32	35 (3)	17.97 (17.46)	8.59 (8.20)	3.68 (3.56)
		EPD	32	34 (2)	5.06 (5.68)	1.78 (1.91)	0.93 (0.96)
		MB	34	34	8.94	3.68	4.25
		LGG	23	23	1.74	0.65	0.33
		CPC	2	2	2.00	1.50	-
		Subtotal	123	128			
	SOLID	MEL	4	4	112.25	51.25	6.00
		NBL	44	47 (3)	15.2 (16.62)	7.09 (7.79)	-
		ACT	20	20	11.75	3.70	1.75
		RHB	14	15 (1)	15.14 (18.00)	6.71 (8.13)	2.08 (3.14)
		OS	27	27	18.22	7.07	2.92
		RB	5	5	5.20	2.40	-
		EWS	19	19	5.63	2.00	-
		Subtotal	133	137			
TCGA		LUAD	129	129	226.63	95.74	36.99
		LUSC	33	33	224.58	95.88	58.06
		SKCM	133	133	411.50	167.57	60.64
		Subtotal	295	295			

^a^The number in the parentheses denotes the number of relapse samples

^b^The number in the parentheses denotes the average number when relapse samples included

### HLA type prediction and validation

Accurate identification of HLA alleles in the patients is essential for patient-specific neoepitope prediction. To select an appropriate algorithm for HLA typing, we compared the performance of OptiType [45] with HLAminer [46] on 51 patients whose HLA alleles were typed in the current study using classic methods including sequence-specific oligonucleotides (SSO), sequence-specific primer (SSP), and Sanger sequence based testing (SBT) technologies (Additional file 2). Consistent with a prior report [47], OptiType achieved higher accuracy (94.1%) than HLAminer (75.5%); we therefore employed OptiType to characterize HLA class I alleles for the entire cohort.

We found that HLA-A*02:01 and HLA-B*07:02 were the most common alleles at HLA-A and HLA-B loci as they were present in 212 (39.3%) and 105 (19.6%) patients, respectively. For HLA-C, the most prevalent alleles were HLA-C*04:01 and HLA-C*07:01 present in 146 (27.0%) and 144 (26.7%) patients, respectively. Comparison of ethnicity projected from HLA-A-B-C alleles with those from genome-wide SNP analysis showed a significant association (*p* < 0.001), indicating the high accuracy of the HLA haplotype prediction.

### Identification of potential neoepitopes based on missense mutations

**Fig. 2:**
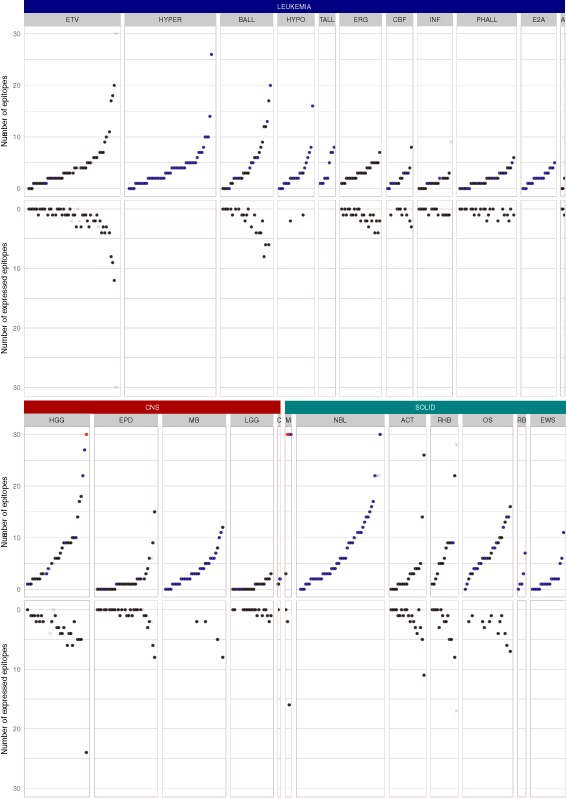
The landscape of neoepitopes in 540 pediatric cancer patients of 23 subtypes. The number of predicted epitopes and expressed epitopes is shown for each sample. The results are shown by the three major cancer types (i.e., leukemia, CNS tumors, and solid tumors) with each of the 23 cancer subtypes shown in a *box*. Within each cancer subtype, the tumor samples are sorted by ascending order of the number of predicted epitopes. The numbers of total epitopes and expressed epitopes are depicted at the *top* and the *bottom mirrored panels*, respectively. The relapse samples are shown as *cross marks* in *grey*. The samples without RNAseq are shown in *blue*. The upper bound is set to 30 and the values > 30 are shown in *red*. Leukemia: *ETV* ETV6-RUNX1 acute lymphoblastic leukemia (ALL); *BALL* B-lineage ALL; *HYPER* hyperdiploid ALL; *HYPO* hypodiploid ALL; *TALL* T-lineage ALL; *ERG* ALL with alterations of ERG; *INF* infant ALL; *CBF* core binding factor leukemia; *PHALL* Ph + (Philadelphia) ALL; *E2A* B-lineage ALL; *E2A* E2A-PBX1 dsubtype; *A* M7 subtype of AML (acute megakaryoblastic leukemia). CNS tumors: *HGG* high-grade glioma; *EPD* ependymoma; *MB* medulloblastoma; *LGG* low-grade glioma; *C* choroid plexus carcinoma. SOLID tumors: *M* melanoma; *OS* osteosarcoma; *NBL* neuroblastoma; *RHB* rhabdomyosarcoma; *ACT* adrenocortical tumor; *RB* retinoblastoma; *EWS* Ewing’s sarcoma

Of the 5619 somatically acquired missense mutations identified in the 540 primary tumors, 2336 were predicted to encode potential neoepitopes that can be bound by at least one of the patients’ HLA alleles with an affinity of < 500 nM (Fig. 2 and Additional file 1). Since neoepitopes must ultimately be validated for their presentation and recognition by T lymphocytes, the use of the term “neoepitope” throughout the text should be read as “potential neoepitope”. The predicted neoepitopes were found in 88.4, 78.1, and 89.8% of leukemia, CNS tumors, and solid tumors, respectively. Leukemias had a median of six missense mutations (range 1–64) with a mean of 3.3 neoepitopes. Sixteen B-lineage acute lymphoblastic leukemia (B-ALL) had ten or more neoepitopes, including five with an ETV6-RUNX1 translocation (ETV), five hyperdiploid B-ALLs, three with intrachromosomal amplification of chromosome 21(iAMP21), one Ph-like, one with IGH-DUX4 translocation and one hypodiploid B-ALL. CNS tumors had a median of five missense mutations per tumor (range 1–98) with a mean of 3.9 neoepitopes, and nine high grade gliomas, three medulloblastoma, and one ependymoma had ten or more neoepitopes. Non-CNS solid tumors had a higher mutation burden (median = 11, range 1–185) with a mean of 7.0 neoepitopes. A total of 27 (20.3%) had ten or more neoepitopes primarily in neuroblastoma (13 cases) and osteosarcomas (seven cases). It is important to note that a single mutation can generate multiple putative neoepitopes by binding to diverse MHC alleles or in distinct registers.

**Fig. 3:**
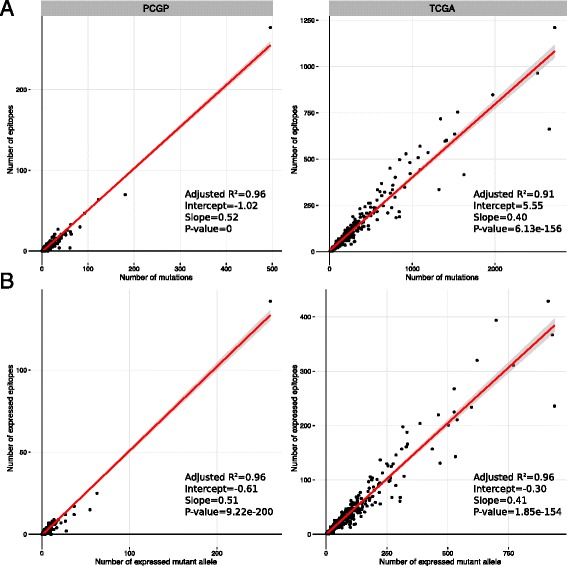
Correlation of mutation burden and the number of (expressed) epitopes in PCGP (*left*) and TCGA (*right*). **a** Regression of mutation burden and number of epitopes in each sample. **b** Regression of number of mutations and number of expressed epitopes in each sample. The *p* value and R^2^ value of the regression are labeled

Approximately half of the primary tumors (*n* = 270) were characterized by transcriptome sequencing (RNA-seq), which allowed us to ascertain the expression status of potential neoepitopes. A total of 2838 missense mutations were identified from the 270 tumors, of which 1180 mutant alleles were expressed (41.6%). The proportion of expressed mutant alleles encoding neoepitopes (37.4%, 441/1180) is comparable to the proportion of total missense mutations encoding neoepitopes (41.6%, 2336/5619). The number of mutations showed a strong linear correlation with the number of neoepitopes (R^2^ = 0.96, *p* value < 0.01). Similarly, the number of expressed mutant alleles was also strongly correlated with the number of expressed epitopes (R^2^ = 0.96, *p* value < 0.01) (Fig. 3). Of the 270 tumors, 163 (60.1%) harbor at least one expressed neoepitope. Four tumors were found with ten or more expressed neoepitopes, including one B-ALL with an ETV6-RUNX1 translocation, one high grade glioma, one melanoma, and one adrenocortical tumor.

Across the pediatric cancer cohort, the proportion of expressed missense mutations encoding neoepitopes is comparable across tumor class, including leukemias (0.38), CNS tumors (0.38), and solid tumors (0.36). Interestingly, melanoma had the highest number of expressed neoepitopes but the lowest proportion (0.29) among tumor types. For the adult TCGA data, we identified 36,230 expressed mutant alleles from 91,375 mutations in 295 tumors. The proportion of expressed mutant alleles encoding neoepitopes was 0.41 (14,753/36,284). Similar to the PCGP data, the number of expressed mutant alleles was strongly correlated with the number of expressed putative neoepitopes (R^2^ = 0.91, *p* value < 0.01) (Fig. 3).

Mismatch-repair deficient cancers have been predicted to have a high number of neoepitopes that might be recognized by the immune system [48]. In the PCGP cohort, four high-grade gliomas (HGG)—SJHGG003_D, SJHGG030_D, SJHGG111_D, and SJHGG034_D—have a relatively high mutation burden (median = 6778, range 224–20,073) (Additional file 1). SJHGG003_D, SJHGG111_D, and SJHGG034_D harbored mutations in DNA mismatch repair genes (PMS2 or MSH6). All of the four hypermutators had ten or more neoepitopes with a mean of 6640 neoepitopes. The proportion of expressed mutant alleles encoding neoepitopes and the proportion of total missense mutations encoding neoepitopes is 38.4% (2797/7290) and 35.3% (11,959/33,853), respectively. We performed mutation signature analyses for the HGG hypermutators along with the four melanoma samples with high mutation burden (Additional file 3). Two major mutation signatures, which correspond to COSMIC signatures 1 and 14, are present in the hypermutators. The mutation signature 1 is correlated with the age of cancer diagnosis; the signature 14 has been observed in samples with high mutation burden [49]. Two out of the three HGG tumors with signature 14 harbor bi-allelic loss-of-function mutations in PMS2, suggesting a potential link between signature 14 and PMS2 mutation. The major mutation signature in the melanoma samples is associated with ultraviolet light exposure [49].

**Fig. 4:**
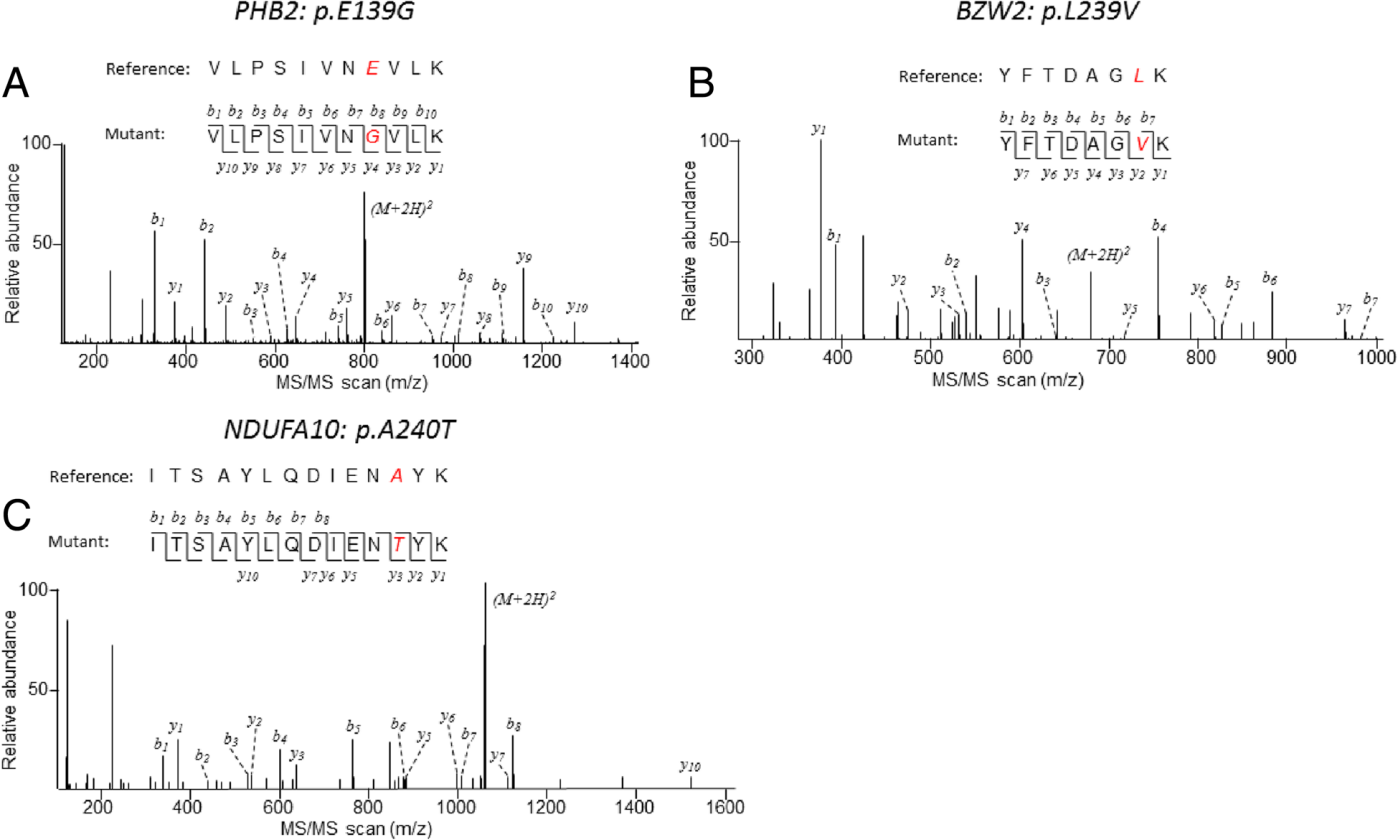
Protein expression of predicted neoepitopes in three rhabdomyosarcoma. For each of the three mutant peptides predicted to be antigenic, the corresponding tandem mass spectrometry (MS/MS) spectra are shown. During each round of MS/MS analysis, ions for the peptide being sequenced were fragmented into complementary ion pairs, with *b*- and *y*- ions corresponding to the N- and C-terminal fragments, respectively (as shown for each mutant peptide sequence, with the mutant amino acid highlighted in *red*). Peaks that match to theoretically calculated fragmented ions of the mutant peptide are indicated. The ions for the peptide itself (precursor ions) are indicated as (*M + 2H*)^2^. **a**–**c** MS/MS spectra assigned to mutant peptides of xenograft samples derived from primary tumors of SJRHB011_E (**a**), SJRHB012_D (**b**), and relapsed tumor SJRHB026_S (**c**)

To provide direct evidence that the predicted neoepitopes were translated and existed at appreciable levels in the cell for antigen presentation, we assessed proteomics data generated from mouse xenografts of three rhabdomyosarcoma tumors. Using the predicted mutant amino acid variant as a marker, we were able to identify peptides corresponding to the mutant antigenic protein in all three samples (Fig. 4), providing further support that these putative epitopes have the potential to be presented by HLA.

### Neoepitopes encoded by recurrent missense mutations

**Fig. 5:**
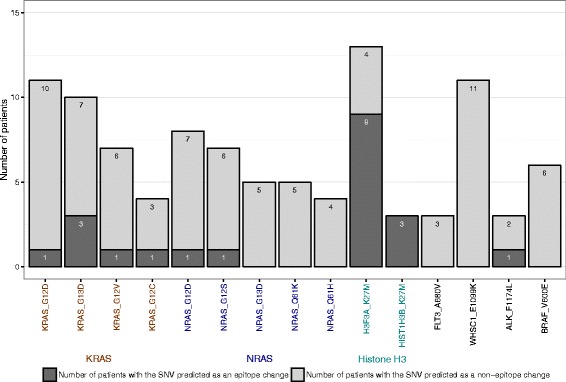
Immunogenicity of recurrent oncogenic missense mutations in pediatric cancer. Somatic missense mutations occurring in tumors from three or more patients were included. *Dark gray* shows the number of samples with the SNV predicted as neoepitopes. *Light gray* indicates the number of samples with no predicted neoepitopes

Across the entire PCGP cohort, we identified 15 recurrent missense mutations present in at least three patients (Fig. 5), all of which are known oncogenic driver mutations. Of these, four KRAS mutations, two NRAS mutations, two histone H3 mutations, and one ALK mutation were predicted to encode epitopes in at least one tumor (Fig. 5; Additional file 4). Notably, the KRAS G13D mutation generated a VVGAGDVGK epitope (285.24 nM) that was predicted to bind the HLA-A*11:01 allele in two hyperploid B-ALLs and one hypoploid B-ALL. The neoepitopes in histone H3 were generated by K27M mutations in HIST1H3B and H3F3A, which share a high degree of protein similarity (96%). The K27M mutations of these two histone H3 genes generated a high affinity neoepitope, ATKAARMSA (4.02 nM), which was predicted to bind the HLA-A*30:01 allele in three high-grade glioma patients, SJHGG008, SJHGG077, and SJHGG004 (Additional file 4). Another two similar neoepitopes from H3 K27M mutations, MSAPATGGV and MSAPSTGGV, were predicted to bind HLA-B*15:17, HLA-A*68:02, HLA-A*02:05, HLA-C*12:03, or HLA-C*03:04 alleles in nine different high-grade glioma patients.

### Neoepitopes derived from gene fusions

**Fig. 6:**
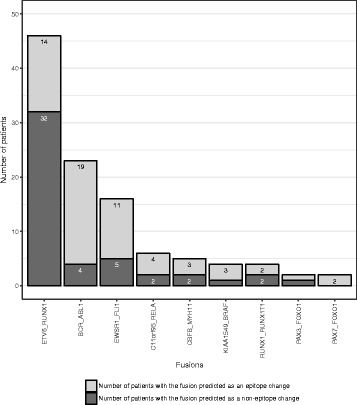
Immunogenicity of recurrent gene fusions in pediatric cancer. *Dark gray* shows the number of samples with the gene fusion predicted as neoepitopes. *Light gray* indicates the number of samples with negative results of neoepitope prediction

To examine neoepitopes generated by gene fusions, we identified the precise junctions of expressed fusion transcripts from RNAseq and predicted neoepitopes from all tiling nonameric peptides overlapping the fusion junction (Fig. 1b). A total of eight distinct gene fusions were found to encode neoepitopes in at least one patient (Fig. 6). Of the 46 B-ALLs with ETV6-RUNX1 fusions, 68% (32/47) were predicted to have neoepitopes resulting from the ETV6-RUNX1 fusion protein. The remaining fusions that generated neoepitopes in multiple cancers included BCR-ABL1, C11orf95-RELA, CBFB-MYH11, EWSR1-FLI1, and RUNX1-RUNX1T1 (Fig. 6).

## Discussion

In the present study, we examined the neoepitope landscape of pediatric cancers based on the somatic missense mutations and gene fusions in tumors sequenced and analyzed through the PCGP. Neoepitopes identified from oncogenic mutations are ideal targets for immunotherapy, including tumor vaccines [50] and adoptively transferred tumor-reactive T cells [51]. Alternatively, checkpoint blockade therapy might facilitate cytotoxic T lymphocyte recognition of these neoepitopes in a subset of patients. Similar approaches may be leveraged to target neoepitopes derived from fusion proteins that are known biomarkers for pediatric leukemias and some solid tumors. To facilitate neoepitope analysis by other research groups, we have deployed our workflow into the cloud under the DNANexus platform to support HLA typing and epitope prediction. These two analyses can be combined into a single workflow under DNAnexus.

The mutation rate in pediatric cancers is low compared to adult cancers [24]. Consequently, the number of predicted neoepitopes per tumor in pediatric cancer (median 2, mean 26.2, range 0–7544) is much lower than those reported in adult cancers (median 112, range 8–610) [24]. A separate analysis using functional and tetramer-binding assays to determine the proportion of these epitopes that elicit responses is in preparation.

Mutations in the DNA mismatch repair genes (MSH2, MSH6, MLH1, PMS2) can lead to high mutation rate and microsatellite instability. Importantly, mutations associated with neoepitopes in DNA mismatch repair-deficient cancers have been shown to be sensitive to immune checkpoint blockade, which is independent of the origin of tissue [48]. The HGG hypermutators in the PCGP cohort with defects in the DNA mismatch repair machinery showed a mean of 8463 mutations per tumor as compared to ten mutations per tumor in the other samples. A mean of 2990 mutations in the hypermutators were found encoding neoepitopes as compared to four in mismatch repair-proficient cancers. The increase in the number of mutations and neoepitopes resulting from mismatch repair deficiency suggests an enhanced immune response in this subset of cancers [52] and is worth further investigation.

A recent study reported that tumor growth in a xenograft tumor model was significantly reduced by adoptive transfer of peripheral blood lymphocytes transduced with T-cell receptors (TCRs) derived from immunized HLA-A*11:01 transgenic mice. These TCRs were highly reactive to the KRAS G12V and G12D mutations [53]. For the PCGP cohort, we found that four distinct KRAS mutations were able to generate putative neoepitopes predicted to be bound by either the HLA-A*11:01 allele (KRAS G13DV and G12D) or the HLA-A*03:01 allele (KRAS G12V and G12C). The HLA-A*11:01 allele was present in 64 patients (12%) in the PCGP cohort; the HLA-A*03:01 allele was present in 110 patients (20%). The high population frequency of the identified HLA alleles and the prevalence of epitopes with predicted high affinity to these HLA alleles suggest that they may be useful targets for future development of immunotherapy.

We additionally identified high affinity neoepitopes encoded by recurrent H3 K27M mutations and ETV6-RUNX1 gene fusions in a high proportion of tumors harboring these somatic alterations. The neoepitopes of histone H3 K27M mutations can be presented mainly by the HLA-A*30:01 allele that is present in 11.9% of African-Americans [36]. The neoepitopes of ETV6-RUNX1 gene fusions can be bound by HLA-A*02:01, which is prevalent in Europeans and US Caucasians (47.8%) as well as other populations. These predicted neoepitopes are potentially important candidates for further immunogenicity testing.

## Conclusions

The repertoire of putative neoepitopes identified in this study (Additional files 5 and 6) provides new fundamental knowledge on the formation of potentially targetable neoepitopes in childhood cancer and will serve as a valuable public resource for development of novel therapeutic strategies against these difficult to treat illnesses. To the best of our knowledge, this is the first comprehensive analysis of neoepitopes in pediatric cancers, which we hope will enable a broader range of research and open up new avenues for the treatment of pediatric cancer.

## Additional files

Additional file 1: Summary of neoepitopes at the sample level. (XLSX 67 kb)

Additional file 2: Accuracy of Optitype HLA typing. (XLSX 66 kb)

Additional file 3: Mutation signature analyses. (XLSX 56 kb)

Additional file 4: Neoepitopes encoded by recurrent missense mutations. (XLSX 48 kb)

Additional file 5: Epitopes predicted from SNVs. (XLSX 300 kb)

Additional file 6: Epitopes predicted from gene fusions. (XLSX 59 kb)
